# The MKK7-MPK6 MAP Kinase Module Is a Regulator of Meristem Quiescence or Active Growth in Arabidopsis

**DOI:** 10.3389/fpls.2019.00202

**Published:** 2019-03-05

**Authors:** Róbert Dóczi, Elizabeth Hatzimasoura, Sara Farahi Bilooei, Zaki Ahmad, Franck Anicet Ditengou, Enrique López-Juez, Klaus Palme, László Bögre

**Affiliations:** ^1^Centre for Systems and Synthetic Biology, School of Biological Sciences, Royal Holloway, University of London, Egham, United Kingdom; ^2^Institute of Agriculture, Centre for Agricultural Research, Hungarian Academy of Sciences, Martonvásár, Hungary; ^3^Institute of Biology II, University of Freiburg, Freiburg im Breisgau, Germany; ^4^BIOSS Centre for Biological Signalling Studies, University of Freiburg, Freiburg im Breisgau, Germany; ^5^Centre for Systems and Synthetic Biology, School of Biological Sciences, University of Freiburg, Freiburg im Breisgau, Germany

**Keywords:** MAP kinase, meristem, Arabidopsis, signaling, de-etiolation

## Abstract

Plant growth flexibly adapts to environmental conditions. Growth initiation itself may be conditional to a suitable environment, while the most common response of plants to adverse conditions is growth inhibition. Most of our understanding about environmental growth inhibition comes from studies on various plant hormones, while less is known about the signaling mechanisms involved. The mitogen-activated protein kinase (MAPK) cascades are central signal transduction pathways in all eukaryotes and their roles in plant stress responses is well-established, while increasing evidence points to their involvement in hormonal and developmental processes. Here we show that the MKK7-MPK6 module is a suppressor of meristem activity using genetic approaches. Shoot apical meristem activation during light-induced de-etiolation is accelerated in *mpk6* and *mkk7* seedlings, whereas constitutive or induced overexpression of MKK7 results in meristem defects or collapse, both in the shoot and the root apical meristems. These results underscore the role of stress-activated MAPK signaling in regulating growth responses at the whole plant level, which may be an important regulatory mechanism underlying the environmental plasticity of plant development.

## Introduction

Organogenesis in plants differs from the process in vertebrates, being mainly postembryonic and continuing throughout the life of the plant. Furthermore, organ growth and morphogenesis in sessile plants show a remarkable plasticity to allow environmental adaptation. “Classical” stress hormones act mainly as growth repressors, while other hormones act as growth promoters. It is increasingly clear that action of the long-established growth factor, auxin, is also strongly influenced by environmental signals (Potters et al., 2007; Kazan and Manners, 2009; Habets and Offringa, 2014). A good example of environmentally induced developmental response is that of the shoot apical meristem (SAM) to light. In flowering plants, etiolated seedlings which germinate in darkness undertake a developmental program called skotomorphogenesis, in which the embryonic stem elongates but leaf growth at the SAM is arrested, i.e., the meristem is in a state of “quiescence”. While de-etiolation of subterranean seedlings emerging into light is a critical event in plant development, the rapid and synchronous induction of growth in shoot apices when dark-grown seedlings are transferred to light (photomorphogenesis) also offers an excellent synchronized experimental system to assess the state of shoot meristem activity. De-etiolation was successfully used to unravel the regulatory program underlying meristem activation in *Arabidopsis thaliana* (López-Juez et al., 2008; Yoshida et al., 2011; Pfeiffer et al., 2016; Mohammed et al., 2018). Remarkably, a number of mitogen-activated protein kinase (MAPK) signaling genes, including MPK6, were identified with high dark expression and rapid light downregulation (López-Juez et al., 2008).

The MAPK phosphorylation cascades are conserved signaling modules in all eukaryotes, consisting of three types of enzymes, which are activated through sequential phosphorylation (Avruch, 2007). In Arabidopsis, genes encoding 20 MPKs and 10 MAPK kinases (MKKs) were identified, and both MPKs and MKKs are classified into four phylogenetic groups, designated A–D (MAPK Group, 2002). Plant MAPKs have been mainly associated with stress signaling, but their role in developmental processes is increasingly evident (Colcombet and Hirt, 2008; Hahn and Harter, 2009; Pitzschke et al., 2009; Rodriguez et al., 2010; Xu and Zhang, 2015).

Although our current knowledge of the intervening MKKs belonging to group D is restricted to two members of this group, MKK7 and MKK9 appear to be of special interest in terms of cross-talk between developmental and stress regulation. MKK9 participates in salt signaling (Alzwiy and Morris, 2007; Xu et al., 2008) and is functionally associated with ethylene biosynthesis and signaling (Xu et al., 2008; Yoo et al., 2008). MKK7 inhibits polar auxin transport (PAT) and promotes pathogen defense and programmed cell death, while expression of the *MKK7* gene is induced by pathogen infection (Dai et al., 2006; Zhang et al., 2007; Popescu et al., 2009; Jia et al., 2016). MKK7 and MKK9 are also involved in stomatal cell fate regulation (Lampard et al., 2009).

Newly formed organs in plants are derived from meristems, the source and organizing tissue of growth. By utilizing light-induced de-repression of etiolated SAMs as a synchronized plant developmental model and using complementary genetic approaches, here we demonstrate a meristem-repressive function of a MAPK pathway, minimally consisting of the MKK7-MPK6 module. Control of meristem activity by environmentally activated, MAPK-mediated signaling represents a novel regulatory mechanism underlying the environmental plasticity of plant development.

## Materials and Methods

### Plant Materials

*Arabidopsis thaliana* Col-0 was used as genetic background. Seeds were germinated on 0.5× Murashige and Skoog (MS) medium (Duchefa), and plants were grown at 21–23°C, 60–70% relative humidity and 140 (±20) μmol m^-2^ sec^-1^ cool white light under long-day (16 h of light/8 h of dark) conditions. The T-DNA insertion lines SM_3_21446, SM_3_21961, and SM_3_36605 for *mkk7* and Salk_073907 for *mpk6* were obtained from the Nottingham Arabidopsis Stock Centre. The insertion sites were verified by cloning and sequencing the PCR products of left-border- and a flanking-sequence-specific primer pairs. Transgenic Arabidopsis lines were generated using the floral dipping method (Clough and Bent, 1998). Inducible MKK7 overexpression lines are viable (Huck et al., 2017; Dory et al., 2018), two independent lines were used in the experiments for this study. The experiments reported here were repeated with at least three independent biological replicates; with similar results.

### Meristem De-Etiolation Assay

The principle of using de-etiolation for assaying SAM activation is described in López-Juez et al. (2008). Following sterilization and stratification, seeds were exposed to light for 30 min to induce germination, and incubated in the dark for 72 h. The etiolated seedlings were subsequently transferred to continuous light and harvested at various time points. Twenty to forty seedlings were measured for each genotype and time point in all experiments. Seedlings were fixed in 90% acetone on ice and washed and stored in 70% ethanol. For microscopic image capture seedlings were mounted in Hoyer’s solution (80 g chloral hydrate, 10 ml glycerol in 30 ml water) before visualization in an Optiphot 2 microscope equipped with a DXM1200 camera (Nikon). For statistical analysis area of emerging leaf primordia were quantified using the ImageJ software (National Institutes of Health, United States). The experiments were repeated three times with *mpk6* and *mkk7* (SM_3_21446) with similar results. In case of *mkk7* the experiment was also carried out with two additional insertion lines (SM_3_21961 and SM_3_36605) with similar results.

### Quantitative Real-Time PCR

Total RNA was isolated using the Qiagen RNaesy Plant Mini Kit (Qiagen), according to manufacturers’ instructions. The optional DNase treatment was also performed using the Qiagen DNase away (Qiagen).

cDNA was synthesized using the Retroscript kit (Ambion) from RNA extracted from 10-day old Col-0 and pK2GW7::MKK7 seedlings (samples collected and pooled from 20 primary transformants). PCR reactions were performed in a Rotor Gene 2000 Real Time Cycler (Corbett Research, Australia), set up with Quantitect SYBR Green PCR Master Mix (Qiagen). Amplifications were performed in duplicate and a control amplification using primers specific for actin was carried out for each run. Primers used: MKK7 F: CCGGAGAGATTTGACTCTGC, R: TTCACGGAGAAAAGGGTGAC, actin: F: GAAGAACTATGAATTACCCGATGGGC, R: CCCGGGTTAGAAACATTTTCTGTGAACG. Gene expression data was calculated by the Delta Ct method.

**FIGURE 1:**
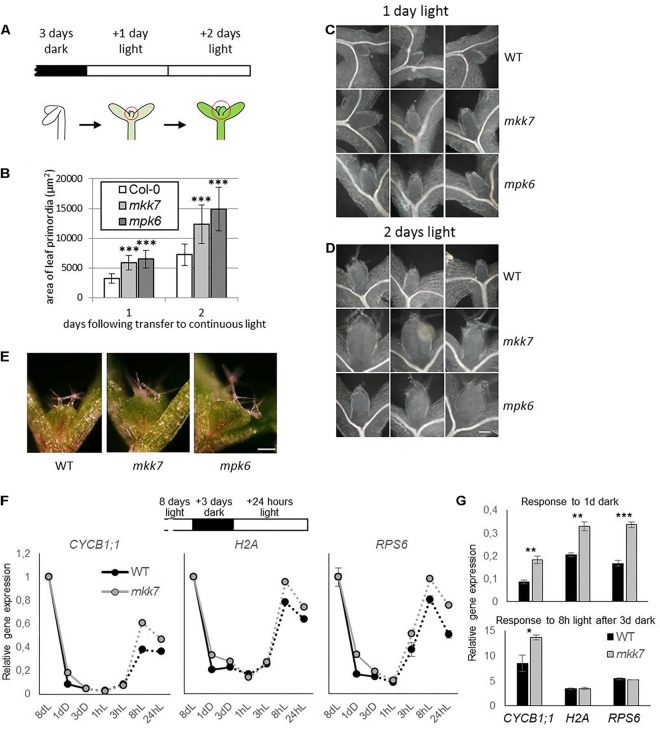
Utilization of seedling de-etiolation as a synchronized developmental model. **(A)** De-etiolation experimental setup. Seeds were germinated for 3 days in darkness then transferred to continuous light. Area of developing leaf primordia were quantified by images of microscopic preparations of seedlings made 1 or 2 days following transfer. **(B)** Area of developing Col-0, *mpk6*, and *mkk7* leaf primordia at 1 and 2 days in continuous light, following 3 days growth in dark. About 30 seedlings were used for each sample. *P*-values: 1.32E-09–2.29E-14, indicated by three asterisks. The error bars represent standard deviation. **(C,D)** Representative images of Col-0, *mkk7*, and *mpk6* leaf primordia at one **(C)** and two **(D)** days following exposure to light. Scale bar: 50 μm. **(E)** Col-0, *mkk7*, and *mpk6* seedlings germinated in darkness for 3 days then exposed to continuous light for further 3 days. Scale bar: 250 μm. The images are representative of at least 12 seedlings. **(F,G)** Relative gene expression of signature genes during dark arrest and subsequent light exposure in emerging leaf primordia. Wild-type seedlings were gown in light on sucrose-containing plates for 8 days, transferred to dark and returned to light after three subsequent days. Two hundred seedlings were harvested for each biological replicate at the corresponding times and had the primordia of leaves 1 and 2 dissected. Gene expression was monitored by quantitative real-time PCR and shown as the entire time course **(F)** and for specific time points relative to that at the onset of dark treatment or at the onset of the light treatment **(G)**. Error bars indicate standard error of the mean (between biological replicates). Asterisks indicate level of significance: ^∗^*p* < 0.05, ^∗∗^*p* < 0.01, ^∗∗∗^*p* < 0.001.

For relative gene expressions of *CYCB1;1, H2A*, and *RPS6* (Figure 1F,G), the experimental setup, sample fixation and shoot apex dissection, RNA isolation, reverse transcription, quantitative RT-PCR, gene-specific primers and data processing were as previously described (Mohammed et al., 2018). First leaf pairs of around 200 seedlings were dissected for each sample and time point under a stereomicroscope. qPCR relative values were determined as amplification efficiency (1.7 or above) to the power of the number of critical threshold cycle.

### Histology and Microscopy

Plant fixation and embedding was done according to (Begheldo et al., 2013). Briefly, plants were fixed with 4% paraformaldehyde in PBS and vacuum infiltrated for 5 min. After fixation, plants were embedded in Paraplast and thin section microscopy was carried out according to Cnops et al. (2006), using 8 μm sections generated with a RM2245 microtome (Leica). Mounted sections were imaged using a Zeiss inverted microscope, images were processed using AxioVision LE software (Zeiss). For visualization of the plant vasculature, seedlings were cleared with 100% ethanol overnight then gradually rehydrated, and stored and dissected in 50% glycerol. Images were obtained using dark field optics on a Zeiss Stemi SV11 Apo stereomicroscope (Carl Zeiss, Göttingen, Germany).

Ethynyl deoxyuridine (EdU), a thymidine analog, kit (Click-iT^TM^ EdU Alexa Fluor^TM^ 488 Imaging Kit) was used to stain S-phase cells in the root meristem. Arabidopsis seedlings were incubated for 1 h in 10 μM EdU-containing liquid MS media, shoots were excised, and the roots transferred to microcentrifuge tubes. Cut-roots were fixed with 3.7% formaldehyde and 0.1% Triton X100 in microtubule-stabilizing buffer (MTSB) under vacuum for 1 h, and subsequently washed with MTSB (3 × 5 min). Samples were then permeabilised with 0.5% Triton X100 in PBS for 15 min at room temperature, and subsequently washed with PBS (3 × 5 min). Next, samples were incubated in the Click-iT reaction mixture for 40 min at room temperature and protected from light, and washed afterwards with PBS (3 × 5 min). EdU-labeled roots were then incubated in 25% of Sysmex CyStain UV Precise P staining buffer which contains 4′,6-diamidino-2-phenylindole (DAPI) in PBS for 15 min at room temperature and protected from light. Finally, samples were washed with PBS (3 × 5 min).

An Olympus IX-81 FV-1000 confocal laser-scanning microscope was used. DAPI, Alexa Fluor 488, and propidium iodide were exited using 405, 488, and 543 nm lasers, respectively, and emitted fluorescence were collected using band pass filters 420–480 for DAPI, 505–530 filter for EdU, and long pass filter 570 for propidium iodide.

### Immunofluorescence Analysis

Samples were fixed and processed as described previously (Galweiler et al., 1998). PIN1 was detected in permeabilised seedlings incubated with an affinity-purified mouse anti-PIN1 monoclonal antibody (1:100) and monoclonal secondary antibody (Alexa 488 goat anti-mouse at 1:1000 dilution). Fluorescent proteins were analyzed with a Zeiss LSM 5 DUO scanning microscope. GFP and DAPI fluorescence were monitored using multi-tracking in frame mode. GFP was excited using the 488 nm laser line in conjunction with a 505–530 band-pass filter. DAPI was excited with the 405 nm laser line and collected using a 420–480 nm band-pass filter.

### Scanning Electron Microscopy

Eight and twelve day old seedlings grown in long-day conditions were used for the scanning electron microscopy (SEM) studies. Seedlings were fixed in 3% glutaraldehyde, 4% formaldehyde, in phosphate buffer (pH 7.2), for at least 2 h and were then washed three times in phosphate buffer (pH 7.2). Samples were gradually dehydrated in 30, 50, 70, 90, and 2 × 100% EtOH. Samples were then critical point dried and then samples were coated with gold by sputter coating. Images were obtained using a Hitachi S-3000N Scanning Electron Microscope.

## Results

### The MKK7-MPK6 Module Is a Negative Regulator of Shoot Meristem De-Repression

We took advantage of the rapid and synchronous induction of growth in shoot apices when dark-grown seedlings are transferred to light, and our previous observation of the rapid, co-occurring repression of several kinase genes, including *MPK6*, to assess the functional significance of MAP kinase signaling in regulating meristem activity. Three-day old dark-grown seedlings were transferred to continuous light to follow the development of leaf primordia. Seedlings were collected at various time points and the surface area of leaf primordia was determined by analyzing microscopic images (Figure 1A). Our results show that the average leaf primordia area in *mpk6* mutants is more than two fold larger than that of control, both 1 and 2 days following exposure to light (Figure 1B–D). Since the upstream MKK7 has a PAT inhibitory function (Dai et al., 2006) we also tested leaf primordia development of *mkk7* seedlings in this setup, with similar results to *mpk6* (Figure 1B–D). The trend of larger leaf primordia in both mutants could be observed even after 3 days of light exposure (Figure 1E).

To gain insight into the role of MKK7 in the dark-induced growth repression at the molecular level we examined the relative expressions of genes associated with mitosis (*CYCB1;1*, encoding Cyclin B1;1), DNA synthesis (S phase) (*H2A*, encoding Histone 2A), and translation capacity (*RPS6*, encoding 40S ribosomal protein S6-1). We have recently demonstrated that a comparable gene expression program to that seen during de-etiolation takes place after an imposed dark arrest, during light reactivation of growth in the emerging leaf primordia (Mohammed et al., 2018). We used this experimental setup to monitor gene expression throughout the dark repression and light de-repression process. Eight-day-old seedlings grown under continuous light were transferred to dark for three days. In line with Mohammed et al. (2018), all three genes were repressed during the 3-day dark period and were up-regulated within 8h following re-exposure to light in both genotypes (Figure 1F). However, a quantitative difference was observed in the kinetics of changes in gene expression between wild type and *mkk7* seedlings. Repression of all three genes was delayed in *mkk7* (Figure 1F), while upregulation of *CYCB1;1*, but not *H2A* and *RPS6* was accelerated (Figure 1G).

These results indicate that MAPK signaling participates in suppression of leaf growth from the SAM under unfavorable environmental conditions (absence of light).

### Increased Expression of MKK7 Impairs Meristem Organization and Leaf Development

**FIGURE 2:**
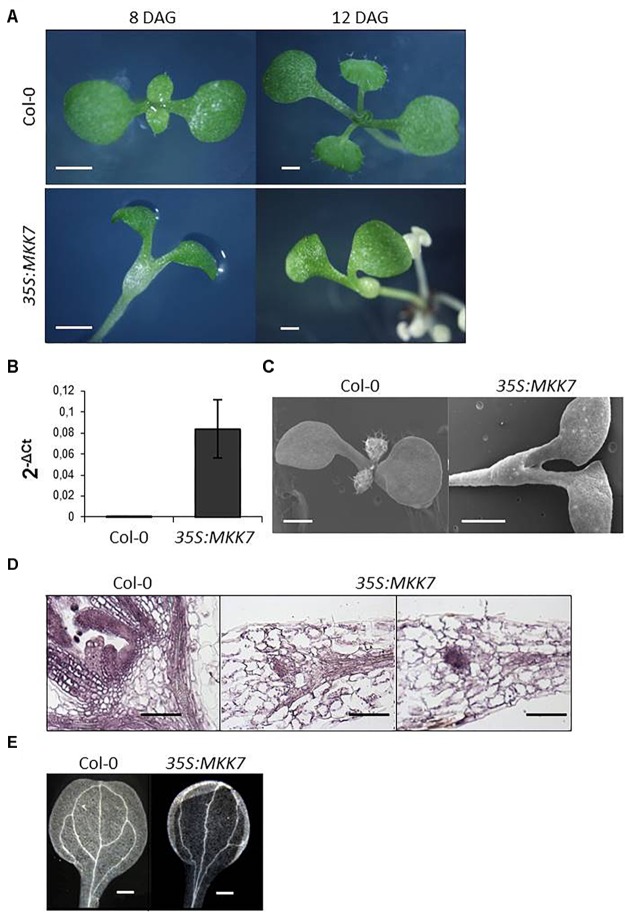
Constitutive overexpression of MKK7 impairs leaf initiation and meristem organization in Arabidopsis. **(A)** Development of Col-0 and *35S:MKK7* seedlings, images are representative of at least 20 seedlings. Images were obtained on a stereomicroscope of Col-0 and *35S:MKK7* seedlings at 8 days and 12 days after germination (DAG) as indicated. Scale bars: 2 mm. **(B)** Detection of transgenic MKK7 expression. qPCR data of MKK7 transcript levels, in Col-0 and *35S:MKK7* seedlings. **(C)** scanning electron microscopic images of 8-day old Col-0 and *35S:MKK7* seedlings, scale bars: 1 mm. **(D)** Longitudinal sections of shoot apices of Col-0 and *35S:MKK7* 8-day old seedlings, scale bars: 200 μm. **(E)** Dark field optics microscopic images showing the vasculature of Col-0 and *35S:MKK7* cotyledons, scale bars: 400 μm. Images are representative of at least five seedlings.

In order to gain further insight into the role of MKK7 in adaptive growth regulation, we attempted to generate plants that overexpress MKK7 tagged with a c-Myc epitope at the N-terminus (*35S:MKK7*). In line with the reported lethality of overexpression of a constitutively active MKK7 version (Meng et al., 2012), kanamycin-resistant *35S:MKK7* primary transformants were not viable, therefore transgene overexpression was assayed in pooled primary transformant plantlets (Figure 2). Intriguingly, we observed severely impaired meristem development of the primary transformant seedlings. The primary transformants failed to initiate true leaves (Figure 2A), failure of organ initiation was confirmed by scanning electron microscopy (Figure 2C). Longitudinal sections along the apical-basal axis (Figure 2D) revealed that *35S:MKK7* seedlings either completely lacked a SAM, or some meristematic-like tissue was observed at the top of the hypocotyl, indicated by small, densely stained cells. Besides the failure to establish normally organized meristems and to properly initiate organs, *35S:MKK7* cotyledons have a simplified vascular pattern missing two of the four loops normally present in the WT cotyledon (Figure 2E).

**FIGURE 3:**
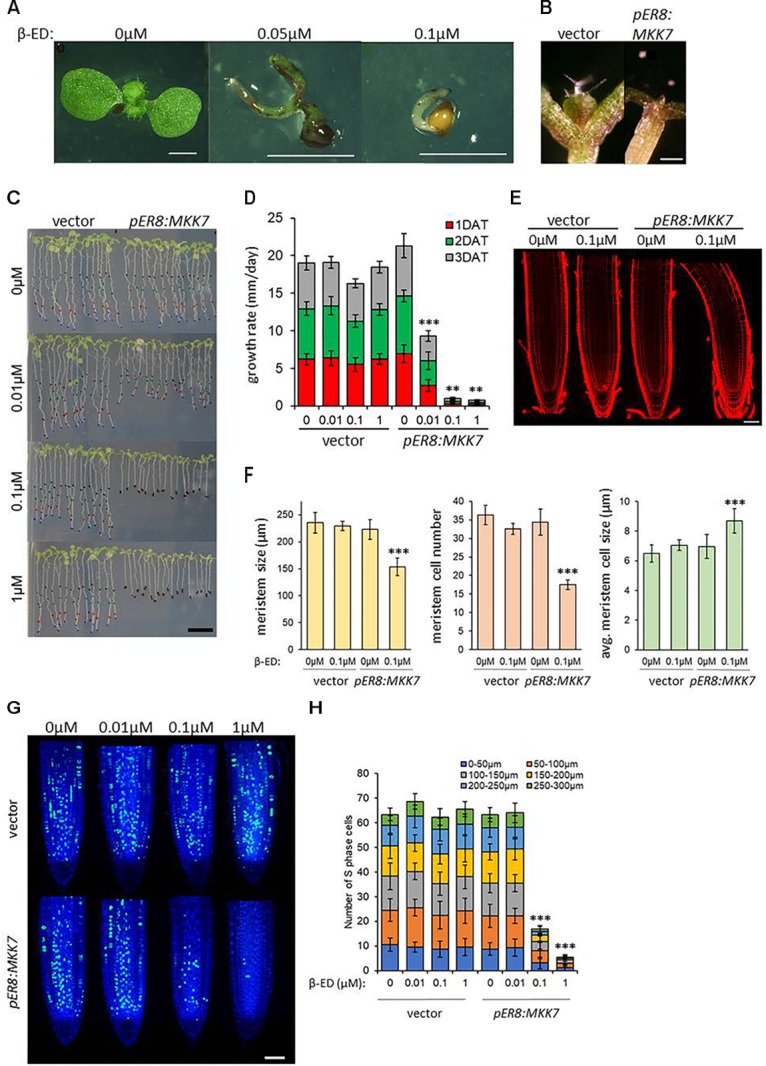
Induced overexpression of MKK7 impairs development and meristem organization in Arabidopsis. **(A)** *pER8:MKK7* (β-estradiol inducible) seeds germinated in the absence or in the presence of 0.05 and 0.1 μM β-estradiol. Scale bars: 2 mm. **(B)** Effect of induced MKK7 overexpression on leaf outgrowth during seedling de-etiolation. Empty-vector transformant and *pER8:MKK7* seedlings were germinated in darkness for three days then transferred to 1 μM β-ED-containing media and exposed to continuous light for further 3 days. Scale bars: 250 μm. **(C,D)** Root growth arrest in response to induced MKK7 overexpression. Six-day old seedlings of empty vector and inducible MKK7 overexpression lines were transferred to β-estradiol containing media at the indicated concentrations. The positions of the root-tips were marked daily for a three-day period. Scale bar = 10 mm. Root growth rate after transfer to β-estradiol-containing growth medium **(D)**. About 40 seedlings were measured for each sample. Error bars indicate standard deviation, asterisks indicate level of significance. **(E,F)** Root meristems at ∼16 h after transfer to ±0.1 μM β-estradiol. Representative images are shown in panel **(E)**, the roots are aligned by the position of the quiescent center (QC). Scale bar = 50 μm. Cell length was measured longitudinally from the quiescent center cells on left-hand side of the root. Root meristem length, number of meristematic cells and average meristematic cell size of empty vector and *pER8:MKK7* lines were measured at ∼16 h after transfer to ±0.1 μM β-estradiol **(F)**. Seven to ten seedlings were measured for each sample. Error bars indicate standard deviation, asterisks indicate level of significance. **(G)** representative micrographs showing cells in S-phase by EdU labeling (green) in the root meristem ∼16 h after transfer to β-estradiol concentrations indicated. Nuclei are counterstained with DAPI (blue). Scale bar = 50 μm. **(H)** Quantification of S-phase cells in the root-tip. Cell counting was performed in 50 μm sections in 300 μm regions upwards from the QC. About 20 roots were quantified for each sample. *T*-tests were performed on the averages of the 50 μm root sections. Asterisks indicate level of significance. Error bars represent standard deviation.

Due to the severity of the phenotypes produced using the constitutive 35S promoter to express *MKK7*, we also generated lines that express *MKK7* under the control of a β-estradiol-inducible promoter system (Huck et al., 2017; Dory et al., 2018). In the absence of β-estradiol all primary transformants developed normally and produced seeds, progeny from two independent homozygous lines was used in subsequent experiments. Transgenic seeds germinated on 0.05 or 0.1 μM β-estradiol gave rise to severely deformed seedlings (Figure 3A). In contrast to the accelerated leaf outgrowth of de-etiolated *mkk7* seedlings, and in accordance to the disturbed SAM organization caused by constitutive overexpression of MKK7, induced overexpression of MKK7 resulted in a severe inhibition and eventual arrest of meristem activation by light during seedling de-etiolation (Figure 3B).

Induced overexpression of MKK7 also led to the rapid arrest of root growth. Six-day old seedlings grown on vertical plates were transferred to β-estradiol-containing media and subsequent root growth was observed for 3 days. Transferring six-day old Arabidopsis seedlings to inducing media plates inhibited root growth by ∼50% at 0.01 μM β-estradiol and led to a complete inhibition at 0.1 and 1 μM β-estradiol within 24 h (Figure 3C,D), strongly suggesting that MKK7 overexpression also affects the root apical meristem (RAM). Induction by 0.1 μM β-estradiol led to meristem shortening by ∼30%, while the number of meristematic cells was reduced by ∼50% (Figure 3E,F). This was paralleled by a slight increase of meristematic cell size.

To further demonstrate that MKK7 negatively regulates cell proliferation as indicated by meristem repression, we carried out EdU labeling at the aforementioned estradiol concentrations at 16 h, a known cell cycle length in the root meristem (Hayashi et al., 2013; Yin et al., 2014). EdU incorporation visualizes active DNA replication, and thus can be used as a marker for cell cycle-driven meristem activity. We counted EdU-positive cells in 50 μm sections from the QC cells to capture the cell cycle dynamics across the meristematic zone. There was no inhibitory effect at 0.01 μM, but a dramatic ∼70% inhibition at 0.1 μM and ∼90% at 1 μM was observed (Figure 3G,H). This implies that MKK7 over-expression prevents the onset of DNA replication (S phase) and thus entry into the cell cycle.

### Polar Auxin Transport Is Established During Shoot De-Etiolation Process

Directional auxin distribution is necessary for the recruitment of stem cells into leaf primordia and for their subsequent development into leaves and depends on the re-distribution of auxin efflux transporters, including PIN1 (Heisler et al., 2005). PIN1 directs auxin flow to converge in the marginal epidermis of developing leaf primordia and PIN1 expression is further detected close to the center of each young primordium, aligned toward the hypocotyl in the emerging provascular cells (Scarpella et al., 2006; Tsugeki et al., 2009). Accordingly, shoot apex de-etiolation is characterized by the transient downregulation of auxin responsive genes (López-Juez et al., 2008). The importance of auxin responses in the arrest/activation of the meristem in the light is also highlighted by observations in tomato (Yoshida et al., 2011). Moreover, we have recently demonstrated the gradual establishment of PAT during the de-etiolation process by using the auxin-responsive promoter, DR5 and by immunolocalization of the auxin transporter, PIN1 (Mohammed et al., 2018).

**FIGURE 4:**
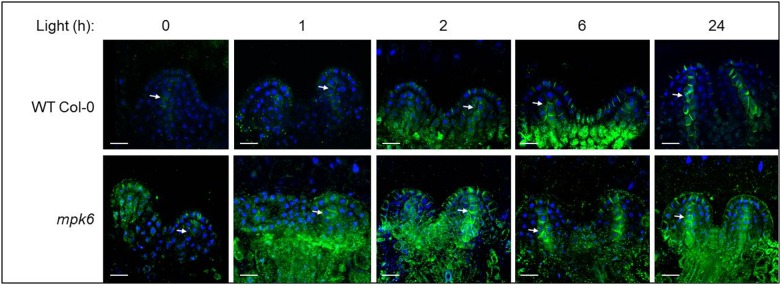
PIN1 expression and localization in developing leaf primordia, in wild type and *mpk6* mutant seedlings, as detected by immunostaining of PIN1. PIN1 (green) and DAPI (blue). Images of the first two leaf primordia of the wild type (top panel) and *mpk6* (bottom panel) during light-induced meristem activation. Seedlings were germinated in the dark for three days, then exposed to continuous white light for the times indicated above (in hours). Arrows indicate midvein. Scale bars: 10 μm. The images are representative of 10 seedlings.

As MPK6-mediated phosphorylation has been implicated in the regulation of PIN1 cellular patterning (Jia et al., 2016; Dory et al., 2018) we decided to examine the role of MPK6 in the establishment of PIN1 pattern. In line with Mohammed et al. (2018), upon transfer to light there is a gradual accumulation of PIN1 in epidermal cells and in the forming midvein. Intracellular distribution of the accumulating PIN1 proteins is mainly polar: apical in epidermal cells and basal in provascular cells. In comparison to wild type, the establishment of the PIN1 distribution pattern is accelerated in the *mpk6* background (Figure 4), implying a negative regulatory involvement of MPK6 in this process.

## Discussion

Due to sessile life style plant development is able to flexibly respond to changing environmental conditions. This developmental plasticity is one of the characteristic differences between animal and plant kingdoms, and it must be orchestrated by integrated environmental and developmental signaling mechanisms. Here we present genetic evidence that the MKK7-MPK6 module participates in meristem regulation driven by a naturally occurring environmental variable.

Light provides an easy-to-manipulate environmental switch for the state of SAM activity and can help to address fundamental questions in meristem function (López-Juez et al., 2008; Mohammed et al., 2018). Along with various other MAPK signaling genes, *MPK6* transcript levels were revealed to be down-regulated within an hour during meristem de-repression, this taking place specifically in the seedling shoot apex, but not in the cotyledons (López-Juez et al., 2008), an especially intriguing finding considering that *MPK6* transcript levels do not vary significantly under most conditions when quantified in whole seedlings (Menges et al., 2008). Therefore, we utilized the light-induced de-repression of the SAM as a tool to survey the activity of the meristem by measuring the expansion rate of developing leaf primordia, and found that MPK6 and the upstream MKK7 function as repressive modulators of organ development from the SAM. Moreover, our results further demonstrate that de-etiolation can be utilized as a synchronized developmental system in plant biology to assess meristem activity, and to analyze the mechanisms controlling leaf initiation and development. In contrast to the accelerated meristem activation in null mutants, constitutive or induced overexpression of MKK7 results in the collapse of meristem organization and a subsequent growth retardation or even full arrest, inducible overexpression demonstrating this to occur in a dose-dependent manner.

Growth of *mkk7* and *mpk6* mutants under standard conditions is wild-type like, single mutants develop normally. In this light, we consider our findings of accelerated de-etiolation remarkable, as in this developmental setting we were able to assign a developmental phenotype to *mkk7* and *mpk6*, which implies their meristem-regulatory functions.

Furthermore, our findings not only demonstrate a developmental impact of this signaling module, they also reveal a biological role, and potentially a fitness value, for this action, in the regulation of quiescence or active growth of the meristem under the control of a natural environmental signal (the first exposure of dark-grown seedlings to light).

These results complement previous findings revealing aspects of developmental regulatory functions of MKK7-MPK6 with a specific role in regulating apical meristems, the central organizing tissues of plant growth.

The CLAVATA (CLV) pathway operates in the regulation of the stem cell population size in the SAM (Dodsworth, 2009). According to the established model a CLV1–CLV2 receptor heterodimer binds the CLV3 peptide ligand. This ligand–receptor interaction leads to the transphosphorylation of the CLV1 kinase domains. Phosphorylated residues of the kinase domain act as binding sites for downstream effector molecules such as kinase-associated protein phosphatase (KAPP) and a Rho GTPase-related protein (ROP). This model is remarkably analogous to the animal growth factor recognition that activates the ERK MAP kinase pathway in response to growth factors and it has been long proposed that the CLAVATA signal transduction could conceivably involve a MAP kinase cascade (Clark, 2001). Indeed, MPK6 activity is controlled by CLV receptors (Betsuyaku et al., 2011), while MAPK-mediated phosphorylation of meristem-regulatory transcription factors has been also demonstrated (Popescu et al., 2009; Dory et al., 2016).

Polar auxin transport and the resulting local auxin maxima sites are important in establishing developmental patterns in plants. PINs determine the direction of PAT through their asymmetric subcellular localization and thus signaling pathways regulating PIN localization can modulate developmental programs in response to triggering stimuli (Robert and Offringa, 2008; Sassi et al., 2012). During leaf initiation auxin maxima mark sites of incipient primordia (Heisler et al., 2005) and leaf venation (Mattsson et al., 2003; Scarpella et al., 2006). The formation of leaf primordia and the emerging vascular cells within are accompanied by the appearance and polar localization of the auxin efflux carrier protein PIN (Tsugeki et al., 2009). In agreement with these findings, a decrease in overall auxin activity during de-etiolation, coupled with the emergence of auxin maxima sites using the *DR5:GUS* reporter line, was found (Mohammed et al., 2018), underscoring the importance of establishing PAT in de-repressed leaf primordia. Leaf primordia tips and pro-vascular cells display particularly strong or emerging auxin response, consistent with auxin drainage being an integral element of the phenomenon of leaf primordia growth (Deb et al., 2015). The MKK7-MPK6 pathway has been demonstrated as a PAT repressor (Dai et al., 2006; Jia et al., 2016) and MPK6-mediated phosphorylation modulates PIN1 cellular localization (Jia et al., 2016; Dory et al., 2018). Therefore we compared the establishment of PIN1 pattern in the emerging leaf primordia in wild type and *mpk6* seedlings and found that this process is accelerated in the absence of MPK6, implying that a difference in auxin drainage can be another regulatory layer underlying the observed acceleration of leaf emergence in this genetic background.

Plants exposed to either abiotic or biotic stress conditions respond by actively altering their growth pattern as part of the overall defense response, which serves to minimize exposure (stress avoidance) and to divert limited resources to defense mechanisms at the expense of growth (Potters et al., 2007). It has been suggested that there is a generic ‘stress-induced morphogenic response’ common to most stresses, which comprises the inhibition of cell elongation, localized stimulation of cell division and alterations in cell differentiation status. This response is regulated by increased reactive oxygen species (ROS) production and altered phytohormone transport and metabolism (Potters et al., 2007). ROS are not only commonly formed during most stresses, they are also well-known MAPK activators. Moreover, redox-regulatory mechanisms are also involved in meristem regulation (Schippers et al., 2016). Remarkably, redox imbalance due to glutathione depletion results in growth reduction and perturbations in both SAM and RAM through inhibition of auxin transport, implying that PIN function is dependent on a post-translational redox regulation (Bashandy et al., 2010; Koprivova et al., 2010).

Taken together, MAPK-mediated meristem regulation is probably highly complex, exerted through the phosphorylation of several substrates. A recent study identified a number of differentially phosphorylated proteins downstream of MKK7-MPK6/3 (Huck et al., 2017), although most of these proteins are defense related, in line with the positive regulatory role of MKK7 in pathogen response (Zhang et al., 2007). However, meristem-derived materials are underrepresented in whole-plant samples and thus are rarely detected by most high-throughput approaches. Detailed characterization of the regulatory network underlying the meristem-regulatory role of stress-activated MAP kinase signaling requires further studies and may unveil an important regulatory mechanism of the environmental plasticity of plant development.

## Author Contributions

EH, EL-J, KP, LB, FD, and RD designed the research. RD, EH, SFB, ZA, EL-J, LB, and FD performed the research. EH, EL-J, KP, LB, FD, and RD analyzed and discussed the data. RD wrote the manuscript with input from all authors.

## Conflict of Interest Statement

The authors declare that the research was conducted in the absence of any commercial or financial relationships that could be construed as a potential conflict of interest.
